# Exploring GNSS Crowdsourcing Feasibility: Combinations of Measurements for Modeling Smartphone and Higher End GNSS Receiver Performance

**DOI:** 10.3390/s19133018

**Published:** 2019-07-09

**Authors:** Ville V. Lehtola, Stefan Söderholm, Michelle Koivisto, Leslie Montloin

**Affiliations:** 1 Finnish Geospatial Research Institute FGI, National Land Survey, PO Box 52, 00520 Helsinki, Finland; 2 ITC faculty, EOS Department, University of Twente, PO Box 217, 7500 AE Enschede, The Netherlands; 3 Airbus Defense and Space, 31400 Toulouse, France

**Keywords:** GNSS, smartphone, receiver error, combination of measurement, crowdsourcing, feasibility

## Abstract

GNSS receiver data crowdsourcing is of interest for multiple applications, e.g., weather monitoring. The bottleneck in this technology is the quality of the GNSS receivers. Therefore, we lay out in an introductory manner the steps to estimate the performance of an arbitrary GNSS receiver via the measurement errors related to its instrumentation. Specifically, we do not need to know the position of the receiver antenna, which allows also for the assessment of smartphone GNSS receivers having integrated antennas. Moreover, the method is independent of atmospheric errors so that no ionospheric or tropospheric correction services provided by base stations are needed. Error models for performance evaluation can be calculated from receiver RINEX (receiver independent exchange format)data using only ephemeris corrections. For the results, we present the quality of different receiver grades through parametrized error models that are likely to be helpful in stochastic modeling, e.g., for Kalman filters, and in assessing GNSS receiver qualities for crowdsourcing applications. Currently, the typical positioning precision for the latest smartphone receivers is around the decimeter level, while for a professional-grade receiver, it is within a few millimeters.

## 1. Introduction

Crowdsourcing possibilities for Global Navigation Satellite Systems (GNSS) increase as the amount of GNSS receivers increases, due to an on-going trend of miniaturizing GNSS receiver components and embedding them into various devices such as mobile phones [1]. There are already several examples of crowdsourcing possibilities for GNSS. Research activities are currently being done to perform jamming detection by collecting and processing GNSS signal condition information from a network of GNSS smartphones or inexpensive sensors [2,3]. Challenges in Arctic navigation may be tackled with a crowdsourcing approach, e.g., ice detection for threat prevention [4]. Furthermore, this work is part of a project aiming to improve weather monitoring in areas not yet covered by sophisticated weather measurement instruments, i.e., a project on tropospheric tomography using smartphone GNSS crowdsourcing.

The number of smartphones equipped with chipset GNSS receivers has been experiencing a strong growth over the last few years. About 80% of the sold GNSS devices are installed in smartphones. From the 2017 GSAmarket report [5], GNSS smartphone receivers are expected to represent more than seven billion devices by 2020. These GNSS crowdsourcing possibilities may, or may not, turn into realities. The least expensive and easiest way to examine the feasibility of the new opportunities is by simulation. These simulations need to be sufficiently accurate in representing realistic situations and therefore must be based on measured data. Especially, it is critical that effective measurement errors be accurately reproduced, for if an error is too large, it directly limits what can be done with the data.

Simulations can be done on various levels starting from the molecular level and the study of electric fields, to the level of individual receiver components [6,7], to the level of pseudoranges [8], and on to the level of navigation solutions where the receiver point position can be represented by a Gaussian distribution [9]. Each of these levels offers a different representation of the reality. Here, we limit the focus to the pseudorange level, that is the output that the receiver yields. More precisely, we eliminate the errors caused by atmospheric effects and the satellite clock by using suitable combinations of measurements. Hence, only the errors that originate from the receiver instrumentation are left for our analysis. This is in contrast to the common idea of using combinations of measurements to remove the effect of the receiver instrumentation, e.g., [10,11,12].

The performance level of the instrumentation determines the level of numerical sophistication that can be used in software methodology. For instance, the quality of the receiver clock dictates whether the navigation solutions should be computed separately for each epoch or whether it suffices to model the offset of that clock [6]. Another example is precise point positioning, for which a sufficiently low level of noise in phase observations is crucial [13]. In other words, the level of errors directly influences the level of sophistication, which can be used in the data processing, which in turn determines the feasibility of different crowdsourcing applications.

Detailed theoretical models of the standard deviation of both the Delay Lock Loop (DLL) and the Phase Lock Loop (PLL) measurement errors are well known [10]. These models can be extended to cover also the Doppler measurement errors [14,15,16] so that both the standard deviation and the bias of Doppler measurement errors are modeled as a function of the Carrier-to-Noise density power ratio (C/N0) and tracking loop architecture and parameters, such as the PLL integration time. These theoretical models are extensively used in assessing the impact of the tracking loop parameters on the code, carrier phase, or Doppler measurements, e.g., for the purpose of designing receivers, and in assessing parameter values when optimizing GNSS receiver architectures. However, these detailed models depend on specific information that is not always available. Information on the receiver architectures and on the tracking loop parameter values for smartphone receivers are generally confidential. Hence, theoretical DLL and PLL error models developed in the literature cannot be used to predict or simulate the DLL and PLL errors for an arbitrary COTS smartphone receiver. This problem is multiplied by the fact that a large crowd of receivers very likely contains several different types of receivers. Hence, the evaluation of receiver-originated errors for individual receivers should not be dependent on a priori information about the design and architecture of these receivers. In other words, there is a need to effectively treat the receiver as a black box.

Field measurements can be used for evaluating receiver instrumentation-based errors in a black box fashion when modeling these errors is not feasible. For DLL and PLL errors, estimation by field measurements is done by selecting an appropriate GNSS measurement combination. As an example, the Code Minus Carrier (CMC) combination is extensively used to estimate the standard deviation of the DLL error, which is dominated by multipath in harsh environments [17]. However, this metric is biased since both ionospheric delay and inter-frequency biases affect CMC measurements [18], and the atmospheric model or dual frequency measurements are needed to eliminate the ionosphere part. Hence, the evaluation of the receiver-originated errors should also be free of any atmospheric modeling, while being available also for single-frequency receivers.

The timing fluctuations of the receiver clock are typically characterized using Allan Variance (AVAR), when the clock is studied as a separate entity [19]. If the clock is considered as a part of the receiver, the stability of different smartphone receiver clocks may be estimated in different conditions (such as static, dynamic, open sky, obstructed areas) [20]. Then, Allan variance is also suitable for fingerprinting GNSS receivers, when used in combination with feature detection methods [21]. However, the burning question here is not related to the environmental factors, nor to the behavior of the receiver in identifiable events. The question is whether some of the data obtained from a receiver are sufficiently noise-free so that they could be exploited for a given crowdsourcing purpose. To this end, the best available performance of the receiver is a key indicator for application feasibility.

In this paper, our main contribution is that we present combinations of measurements for error modeling that can be used to estimate the quality of arbitrary GNSS receivers without atmospheric corrections, nor dual frequency measurements, nor the knowledge about the position of the receiver antenna or its architecture. The receiver is effectively treated as a black box and is assessed via field measurements only, which is a convenient prerequisite when processing crowdsourced data from an anonymous receiver. In detail, we study the best available performance of the receivers in navigation space, i.e., the lower bounds of noise in clock drift and offset, the Delay Lock Loop error (DLL), and the Phase Lock Loop error (PLL). Our work builds heavily on the previous studies, but to our best knowledge, the trinity of combinations and the error models that we present go beyond the ones in the existing literature. For instance, the time series that we use to estimate the low bound for the clock drift do not contain multipathing effects or notable features (see Section 3), which, in contrast, are of primary interest when studying clock stability in different environments [20] or receiver fingerprinting [21]. Furthermore, we extend the use of the CMC time difference metric to develop DLL low-bound error models, while the CMC time difference is a zero-mean metric extensively used for another purpose, namely large code multipath error detection [22,23]. In addition to describing how to measure the errors, we derive models to simulate these errors that can be used to examine the feasibility of crowdsourcing applications. Furthermore, we provide exemplary parameters to these error models from a field campaign, so that the quality of recent single-frequency multi-constellation (GPS L1, Galileo E1, GLONASS L1) smartphone GNSS receivers can be compared against the quality of higher end GNSS receivers.

This paper is written in an introductory manner for a two-fold reasoning. First, the abundance of GNSS receivers has led to them being studied and used by many scholars and engineers. We want to extend our message to a broad audience that is outside the traditional field of navigation, including robotics and mobile mapping, and also to industry. Second, it is highly likely that the development in GNSS receiver technology will continue and that the receivers will keep coming out in various designs, meaning that there will be a continuous need to perform new measurements for new devices. The paper is organized as follows. We lay out the steps to perform these measurements of errors and to model them in Section 2. In Section 3, we describe the field campaign and the data used for the numerical analysis. The error model parameters and the validation of the proposed method is presented in Section 4. After a discussion in Section 5, we conclude the paper.

## 2. Methods for Measuring and Modeling Errors of a GNSS Receiver

### 2.1. Receiver Functionality and the Correlation of Some Errors

In order to obtain observables, a GNSS receiver generates a code and a carrier replica signal using application-specific hardware blocks, so-called Numerically-Controlled Oscillators (NCOs). These NCOs are essentially incremental counters and use the receiver clock crystal, typically a TCXO (temperature controlled crystal oscillator)or an OCXO (oven controlled crystal oscillator), to generate a signal that matches the incoming signal from the satellites. Then, the so-called correlators are implemented in software or hardware to generate the amplitude of the correlation function between the incoming signal and the replica generated in the receiver.

The output from these correlators is provided to code and carrier discriminators that in turn will generate an error estimate, i.e., how much is the difference between the incoming signal and the replica. These errors are then filtered and used as a control input for the NCOs. The loop filters are often referred to as the DLL (Delay Lock Loop) used for controlling the code NCO and the PLL (Phase Lock Loop) used for controlling the carrier NCO. If the replica is found to be slightly delayed, the NCO speed will be increased and vice versa.

All of these components, the NCOs, correlators, discriminators, and loop filters, form a closed-loop system for tracking each signal received from the satellites. Each signal has its own set of NCOs, correlators, discriminators, and loop filters, but the receiver only has one clock that provides the reference for all the NCOs. All the above components will contribute to the overall error in the observables. The NCO will for example have quantization errors due to their discrete nature. This error depends largely on the sampling frequency. A higher sampling frequency results in a smaller error. The DLL and PLL filters have a predetermined bandwidth and update rate, which both affect the size of the error in the replica alignment. The lower the bandwidth is and the higher the sampling frequency is, the more accurate the filters are.

In this text, we refer to the DLL and PLL error as the sum of the errors from all the above components, except the clock. The PLL and DLL errors are to a large extent independent of each other, i.e., the errors in these two channels are uncorrelated. The clock, however, is common for all NCOs, and its errors are therefore highly correlated over all channels.

### 2.2. Measurements

The observations of the receiver consist of pseudorange and carrier phase measurements. The pseudorange $\rho =c({\hat{t}}_{Rx}-t_{Tx})$ is a virtual distance (in meters) between the satellite and the receiver, where ρ depends on the receiver time ${\hat{t}}_{Rx}$ and transmission time of the signal denoted by $t_{Tx}$ (note the different time coordinates) and *c* is the speed of light. In detail:

(1) $$\rho^{s_{j}}=\rho_{0}^{s_{j}}+c\cdot dt+\eta_{\rho }^{s_{j}}\;,$$

where the geometric range between the receiver and the satellite $s_{j}$ is denoted by $\rho_{0}^{s_{j}}$, while dt and $\eta_{\rho }^{s_{j}}$ stand for the receiver clock offset and the sum of code measurement errors with respect to satellite $s_{j}$, respectively. For the carrier phase observations, we have (in meters) [24]:

(2) $$\phi^{s_{j}}=\rho_{0}^{s_{j}}+c\cdot dt+\lambda A^{s_{j}}+\eta_{\phi }^{s_{j}}$$

where λ, $A_{\phi }$, and $\eta_{\phi }^{s_{j}}$ are the carrier wavelength, the phase ambiguity, and the sum of carrier measurement errors with respect to satellite $s_{j}$, respectively.

For this work, the unknowns of interest are the DLL-related code noise within $\eta_{\rho }$, the PLL-related phase noise within $\eta_{\phi }$, and the receiver-originated part of the clock offset dt.

### 2.3. Combinations of Measurements

Measuring the pseudoranges $\rho^{s_{j}}$ or the carrier phases $\phi^{s_{j}}$ does not, per se, let us determine the quality of the receiver, which is our ultimate goal. This is because the instrumental, atmospheric, satellite-based, and environmental errors are all contained within the same measurement observables. In order to separate these errors, we look into the combinations of measurements and introduce the concepts of correlated and uncorrelated errors. Note that in Equations (1) and (2), the correlated error, $\epsilon_{correlated}=c\cdot dt$, is the same for both carrier and code and for all channels, whereas the uncorrelated errors, $\eta_{\rho }^{s_{j}}$ and $\eta_{\phi }^{s_{j}}$, depend on the satellite $s_{j}$. This idea goes beyond the standard combinations that are well known in the literature, e.g., the two satellites’ difference [10,11,12], but has an opposite interest. We want to determine the receiver properties, while the interest in the two satellites’ difference is in removing the receiver dependency for relative positioning purposes (see Ch. 21.5.2 in [10]).

### 2.4. Clock Offset

Calculating navigation solutions from Global Navigation Satellite Systems’ (GNSS) signals always requires time synchronization between transmitter and receiver clocks. If the receiver clock has a limited oscillation stability, the offset between the receiver clock and the system time needs to be re-estimated for each observation epoch or eliminated by processing differences between simultaneous observations. On the other hand, when the receiver clock has a high oscillation stability, the offset between the receiver clock and the system time can be modeled [6].

The navigation solution in a GNSS receiver is typically obtained using a Least Squares Estimator (LSE) or more advanced filtering techniques like the Kalman filter. Using these advanced filters allows for the use of a prediction step in the state estimation, which is especially useful when data from multiple sensors are combined to assist in the positioning. Here, as we only use data from a single GNSS receiver at a time, we retain the simpler LSE approach. In order to obtain maximal precision for our clock offset estimate, we rely on the more accurate carrier phase measurements and, thus, actually estimate the clock offset through the velocity LSE using clock drift, $d\dot{t}$, which is the time derivative of the clock offset. For a discussion on different techniques, see [21].

To obtain the clock drift for each constellation at a discrete time $t_{k}$ (k=1,2,3,…), we write the Doppler term:

(3) $$\delta \phi^{s_{j}}\left(t_{k}\right)=\phi^{s_{j}}\left(t_{k}\right)-\phi^{s_{j}}\left(t_{k-1}\right),$$

which eliminates biases that are very slowly varying with respect to time (such as atmospheric delays) and use a simple LSE:

(4) $$v^{2}\left(t_{k}\right)=\sum_{s_{j}}{\left(\delta \phi^{s_{j}}\left(t_{k}\right)-\delta \phi_{0}^{s_{j}}\left(t_{k}\right)\right)}^{2}.$$

to solve the unknowns $\left(v_{x},v_{y},v_{z},d{\dot{t}}_{GPS},d{\dot{t}}_{GLONASS},d{\dot{t}}_{Galileo}\right)$, where *v* stands for velocity. In Equation (4), the expectation terms $\delta \phi_{0}^{s_{j}}$ are calculated for a static receiver and moving satellites. To this end, receiver position is obtained from the standard position LSE calculated using pseudo-ranges, and satellite movement is obtained from the navigation message. Intuitively, if the receiver would be error-free, we would obtain $v^{2}=0$ from Equation (4), since the receiver is kept static during the measurement. However, as the receiver is not error-free, we obtain time-series for the clock drift.

The receiver clock imposes the same error on all clock drifts regardless of the constellation. Therefore, for simplicity, we write $d\dot{t}=d{\dot{t}}_{GPS}$. We assume that the constellation clock offsets develop slowly in time and that we can divide the time series of $d\dot{t}$ into a slowly-varying and a rapidly-varying component:

(5) $$d\dot{t}=d{\dot{t}}^{slow}+d{\dot{t}}^{fast}\;,$$

by using a fourth-order polynomial to represent the slow component. The assumption that the slow component can be represented and subtracted in this way to separate the fast component is something we will assess later in the Results Section (Figure 3).

The satellite clocks, which are atomic, exhibit a substantially slower clock drift than the one in a GNSS receiver. We do not need to use the precise corrections for satellite clocks, because the time difference of Equation (3) cancels out almost all of the satellite clock offset, and the remaining (slow) satellite clock drift is contained within the slow component of Equation (5).

The receiver clock offset dt, our goal, can be obtained with the help of $d{\dot{t}}^{fast}$. If we assume that each measurement $d{\dot{t}}_{i}^{fast}$ is independent, we can write:

(6) $$\mathrm{Var}\left[d{\dot{t}}^{fast}\right]=\frac{1}{N}\sum_{i=0}^{N}{\left(d{\dot{t}}_{i}^{fast}-d{\dot{t}}_{i-1}^{fast}\right)}^{2}=\sigma_{d\dot{t}}^{2},$$

where $\sigma_{d\dot{t}}^{2}$ is the variance of the clock drift caused by the receiver instrumentation. Importantly, Equation (6) utilizes time series of an atmosphere and antenna position-independent observable, $d{\dot{t}}^{fast}$, from which $\sigma_{d\dot{t}}^{2}$ is determined for each receiver (see the Results Section).

The clock offset model can be constructed using the variance of the clock drift, $\sigma_{d\dot{t}}$ of Equation (6), as follows:

(7) $$\left[\begin{array}{c} dt\\ d\dot{t} \end{array}\right]\left(t_{k}\right)=\left[\begin{array}{c} dt\left(t_{k-1}\right)+\Delta t\;\;d\dot{t}\left(t_{k-1}\right)\\ 0 \end{array}\right]+N\left(\left[\begin{array}{c} 0\\ 0 \end{array}\right],\left[\begin{array}{ccc} 0 & & \\ & & \sigma_{d\dot{t}}^{2} \end{array}\right]\right),$$

where the zero-mean Gaussian noise is added to the clock drift and Δt is the time step between receiver observations at times $t_{k}$ and $t_{k-1}$ (typically 1 s). It is noteworthy that Equation (7) reproduces a random walk behavior for the receiver clock offset. More details on clock modeling may be sought from [25] and the references therein.

### 2.5. Delay Lock Loop

For DLL error estimation, we used the same technique of separating the time series into a fast and a slow component as for the clock offset in Section 2.4. The uncorrelated errors for the code pseudorange, ρ, for the signal from satellite $s_{j}$ are written as:

(8) $$\eta_{\rho }^{s_{j}}=\eta_{DLL}^{s_{j}}+\eta_{multipath,\rho }^{s_{j}}+\eta_{atm,\rho }^{s_{j}}+\eta_{constellation,\rho }^{s_{j}}$$

where $\eta_{DLL}^{s_{j}}$ is the error from DLL, $\eta_{multipath,\rho }^{s_{j}}$ is the error from signal multipathing, $\eta_{atm,\rho }^{s_{j}}$ is the error from the atmosphere, and $\eta_{constellation,\rho }^{s_{j}}$ contains satellite-originated errors. The uncorrelated errors can further be divided into a group of slowly-varying errors:

(9) $$\eta_{slow,\rho }^{s_{j}}=\eta_{atm,\rho }^{s_{j}}+\eta_{constellation,\rho }^{s_{j}}$$

and a group of fast-changing errors:

(10) $$\eta_{fast,\rho }^{s_{j}}=\eta_{DLL}^{s_{j}}+\eta_{multipath,\rho }^{s_{j}}\;.$$

Similarly, for the carrier phase, we can write:

(11) $$\eta_{\phi }^{s_{j}}=\eta_{PLL}^{s_{j}}+\eta_{multipath,\phi }^{s_{j}}+\eta_{atm,\phi }^{s_{j}}+\eta_{constellation,\phi }^{s_{j}},$$

where $\eta_{PLL}^{s_{j}}$ is the PLL error (that will cancel out here, but is addressed in detail in Section 2.6), $\eta_{multipath,\phi }^{s_{j}}$ is the error from signal multipathing, $\eta_{atm,\phi }^{s_{j}}$ is the error from the atmosphere, and $\eta_{constellation,\phi }^{s_{j}}$ contains satellite-originated errors. Then, we divide the errors into slowly-varying and fast-changing errors:

(12) $$\begin{array}{cc} \eta_{slow,\phi }^{s_{j}} & =\eta_{atm,\phi }^{s_{j}}+\eta_{constellation,\phi }^{s_{j}} \end{array}$$

(13) $$\begin{array}{cc} \eta_{fast,\phi }^{s_{j}} & =\eta_{PLL}^{s_{j}}+\eta_{multipath,\phi }^{s_{j}} \end{array}$$

Taking the difference between the carrier phase and pseudorange from Equations (8) and (11) yields the following equation:

(14) $$\eta_{\rho }^{s_{j}}-\eta_{\phi }^{s_{j}}=\eta_{slow}^{s_{j}}+\left(\eta_{DLL}^{s_{j}}-\eta_{PLL}^{s_{j}}\right)+\left(\eta_{multipath,\rho }^{s_{j}}-\eta_{multipath,\phi }^{s_{j}}\right)$$

where the slow-varying errors can be combined into one single slowly-varying error. Finally, we make use of the notation $\Delta_{t}\left(f\left(t_{k}\right)\right)=f\left(t_{k}\right)-f\left(t_{k-1}\right)$ for a discrete time series $f(t_{k})$ and calculate the rate of the errors using finite time differences as:

(15) $$\begin{array}{ll} \Delta_{t}\left(\eta_{\rho }^{s_{j}}-\eta_{\phi }^{s_{j}}\right) & =\Delta_{t}\left(\eta_{DLL}^{s_{j}}-\eta_{PLL}^{s_{j}}\right)+\Delta_{t}\left(\eta_{multipath,\rho }^{s_{j}}-\eta_{multipath,\phi }^{s_{j}}\right)+\Delta_{t}\left(\eta_{slow}^{s_{j}}\right)\\ & \approx \Delta_{t}\left(\eta_{DLL}^{s_{j}}+\eta_{multipath,\rho }^{s_{j}}\right)+\Delta_{t}\left(\eta_{slow}^{s_{j}}\right) \end{array}$$

where simplifications are made since $\Delta_{t}\left(\eta_{DLL}^{s_{j}}\right)\gg \Delta_{t}\left(\eta_{PLL}^{s_{j}}\right)$ and the PLL multipath error disappears since it is effectively the error in the Doppler, which is almost unaffected by multipath.

The Delay Lock Loop (DLL) and code multipath noise are obtained by calculating the difference between the pseudorange rate and carrier phase rate observations using Equations (1), (2), and (15), as:

(16) $$\Delta_{t}\left(\rho^{s_{j}}-\phi^{s_{j}}\right)≃\Delta_{t}\left(\eta_{DLL}^{s_{j}}+\eta_{multipath,\rho }^{s_{j}}\right)+\Delta_{t}\left(\eta_{slow}^{s_{j}}\right),$$

where $s_{j}$ denotes the index number of the satellite, and here, $s_{j}$ is chosen to represent the strongest satellite signal (highest signal-to-noise ratio $C/N_{0}$) for the specific receiver during the measurement. Note that the clock offset dt is naturally canceled out by the combination of measurements of Equation (16). The term $\Delta_{t}\left(\eta_{slow}^{s_{j}}\right)$ can be separated by using a polynomial fit (as in Section 2.4) and removed because the rate of change of the slowly-varying error is very small (this is shown in the Results Section). The DLL and multipath noise are further separated by our experimental setup, i.e., we performed experiments in an open sky environment. This allows us to obtain results without multipathing effects.

Ultimately, we can say that the combination of measurements of Equation (16) represents the sum of DLL and code multipath noise. This error can be modeled by assuming that the consecutive measurements with respect to time are independent and from the same normal distribution, i.e., no multipathing. We write:

(17) $$\begin{array}{ll} \Delta_{t}\left(\rho^{s_{j}}-\phi^{s_{j}}\right) & ≃\Delta_{t}\left(\eta_{DLL}^{s_{j}}\right)\\ & ≃N\left(\mu_{DLL},\sigma_{DLL}^{2}\right)-N\left(\mu_{DLL},\sigma_{DLL}^{2}\right)=N\left(0,2\sigma_{DLL}^{2}\right). \end{array}$$

where the carrier phase ambiguity is effectively canceled by the first-order time difference and $\sigma_{DLL}^{2}$ is the variance of the DLL error. We obtain a Gaussian model:

(18) $$\eta_{DLL}^{s_{j}}=N\left(0,\sigma_{DLL}^{2}\right).$$

Importantly, Equation (17) lays out the atmosphere and antenna position-independent observable used in the Results Section to determine the model parameter $\sigma_{DLL}$. The mean of the fast component is effectively zero. Note that actually the variance of the sum of independent random variables is equal to the sum of these variances, so there is no need to assume normal distributions for the purpose of extracting the variance from Equation (17). However, for error modeling purposes, the normal distribution of Equation (18) is a convenient choice, which shall be verified in the Results Section.

### 2.6. Phase Lock Loop

To obtain the PLL error, we calculated the difference between two channels that were from different satellites, i.e., here we used the two satellites’ difference mentioned in Section 2.3,

(19) $$\phi^{s_{i}}-\phi^{s_{j}}=\rho_{0}^{s_{i}}-\rho_{0}^{s_{j}}+\left(\epsilon_{uncorrelated,\phi }^{s_{i}}-\epsilon_{uncorrelated,\phi }^{s_{j}}\right)$$

where the correlated error is again removed as it is the same for both channels. Next, we used time differences to eliminate the true ranges, i.e., the difference between two consecutive observations is as follows:

(20) $$\Delta_{t}\left(\phi^{s_{i}}-\phi^{s_{j}}\right)=\Delta_{t}\left(\eta_{PLL}^{s_{i}}-\eta_{PLL}^{s_{j}}\right)+\Delta_{t}\left(\phi^{slow}\right)$$

Here again, the two different terms for multipath in the phase observations could exist and were neglected. What remains to be shown in the Results Section is that the rate of the change for the slow component, $\Delta_{t}\left(\phi^{slow}\right)$, was actually so small, that it could be omitted. In fact, the slowly-varying error shall be removed using a fourth-degree polynomial.

The PLL error, our goal, was obtained by assuming that consecutive measurements were independent observables, but taken from the same normal distribution and that the different form of the measurements was not satellite dependent, namely:

(21) $$\Delta_{t}\left(\phi^{s_{i}}-\phi^{s_{j}}\right)∼N\left(0,4\sigma_{PLL}^{2}\right).$$

Here, Equation (21) is the atmosphere and antenna position-independent observable used in the Results Section to determine $\sigma_{PLL}$. Note that the factor of four in Equation (21) for the PLL differs from that of Equation (17) for the DLL, because Equation (21) follows from the first-order finite differences with respect to both time and the satellite, i.e., from:

(22) $$\eta_{PLL}^{s_{i}}-\eta_{PLL}^{s_{j}}∼N\left(0,2\sigma_{PLL}^{2}\right)$$

and:

(23) $$\eta_{PLL}^{s_{i}}∼N\left(\mu_{PLL},\sigma_{PLL}^{2}\right),$$

where $\mu_{PLL}\approx 0$, as will be shown in the Results Section, and Equation (23) defines $\sigma_{PLL}$ as a model parameter. Note again that actually, the variance of the sum of independent random variables is equal to the sum of these variances, so there is no need to assume normal distributions for the purpose of extracting the variance in Equation (22). However, we used a normal distribution to model the PLL error in Equation (23) and verify the validity of this choice in the Results Section.

### 2.7. Temporal Correlations

For modeling purposes, it is of interest to see whether the fast component data contain some time-dependent patterns. A natural mathematical way to explore the temporal behavior of a sample is to compute an autocorrelation function for the observation time series. Let $\left\{a_{k}\right\}$ be a time series of real valued data with length *N*. The autocorrelation function *f* of this sequence is written as:

(24) $$f\left(k\right)=\sum_{i=k+1}^{N}a_{i}a_{i-k},$$

where the time lag is expressed as *k* time steps. For our GNSS receiver data, the length of one time step δt was about 1 s.

Winkel [9] simulated the thermal noise effect for DLL and PLL, suggesting that the autocorrelation function $f_{AC}$ for these would be of the form:

(25) $$f_{AC}\left(\tau \right)\propto e^{-\omega \tau }\left(A\cos(a\tau )+B\sin(b\tau )\right)$$

where A,a,B,b, and ω are constants and τ is the time lag. We, in contrast, argue that the temporal correlations should not be related directly to DLL and PLL, but to the receiver clock, because the clock is the common component for the NCOs on which DLL and PLL depend. This discussion continues in the Results Section.

## 3. Experiments and Data

**Figure 1:**
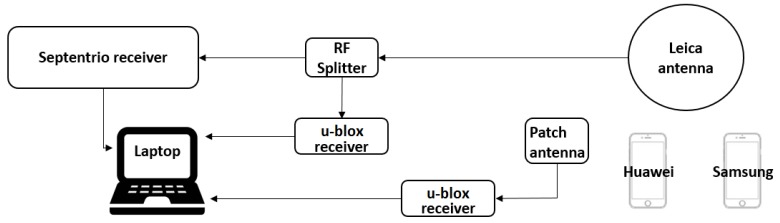
The test schematic. The signal from the Leica antenna was transmitted through the RF splitter to both the u-blox and Septentrio receivers.

In this paper, we consolidated the results from one open sky experiment, although we performed a larger experimental campaign for validation purposes that included two open sky and one semi-urban experiment. The test equipment schematic is shown in Figure 1. A professional-grade Septentrio POLARX5S receiver was connected to a professional-grade Leica AX1202 antenna. Two mass-market u-blox EVK-M8T receivers were connected onto two different antennas, one to the professional-grade Leica antenna and the other one to a mass-market u-blox NEO-M8T patch antenna. Two multi-constellation single-frequency receiver (GPS (L1), Galileo (E1), and GLONASS (L1)) smartphones were employed, Samsung S8 and Huawei P10, which both log raw GNSS data by using the Geo++ RINEX Logger. For processing the data in RINEX files, we used the FGI-GSRx software receiver [26]. Open source software receivers exist, as well, e.g., [27,28,29].

**Figure 2:**
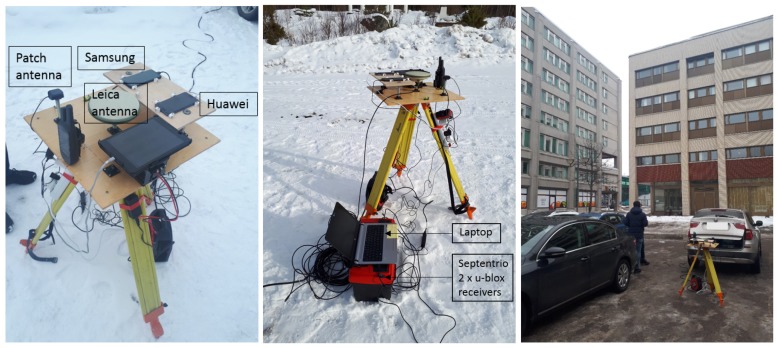
Images from the experimental open sky (**left** and **middle**) and semi-urban (**right**) test campaigns. In all campaigns, the same test equipment setup was used. The two smartphones and the antennas were fixed on a wooden plate.

The same test equipment setup was applied in all three field experiments conducted near Helsinki, Finland; see Figure 2. The open sky experimentation campaign providing the data that we analyzed in this paper was performed in Kirkkonummi (N 604° 9′ 3.7902″, E 24° 32′ 6.7806″). Data were logged for one hour, as was the case for the other two campaigns, as well. The additional open sky experimentation campaign was performed in Metsähovi (N 60° 13′ 02.8400″, E 24° 23′ 40.200″) a few meters away from the permanent FinnRef reference station (FIN18). The semi-urban experiment took place in the city center of Espoo (N 60° 12′ 21.2642″, E 24° 39′ 15.4588″), where some buildings surrounded the test site; see Figure 2. Because of the buildings, some signal multipathing errors were expected at this site.

Note that as we are interested on the low bounds of errors, i.e., the best available performance of the receivers, we do not blindly process the measured time series as is. Instead, a sufficiently long time series of data that do not contain notable features are used for the calculation of results.

## 4. Results

The results for the clock, the DLL, and the PLL models are presented in this order. We present the data from the combinations of measurements of Equations (6), (17), and (21) and concurrently assess how well the models of Equations (7), (18), and (23) proposed in the Methods Section can model this data, e.g., for simulator purposes. All estimated model parameters are gathered into Table 1, which is discussed last.

### 4.1. Clock Model

**Figure 3:**
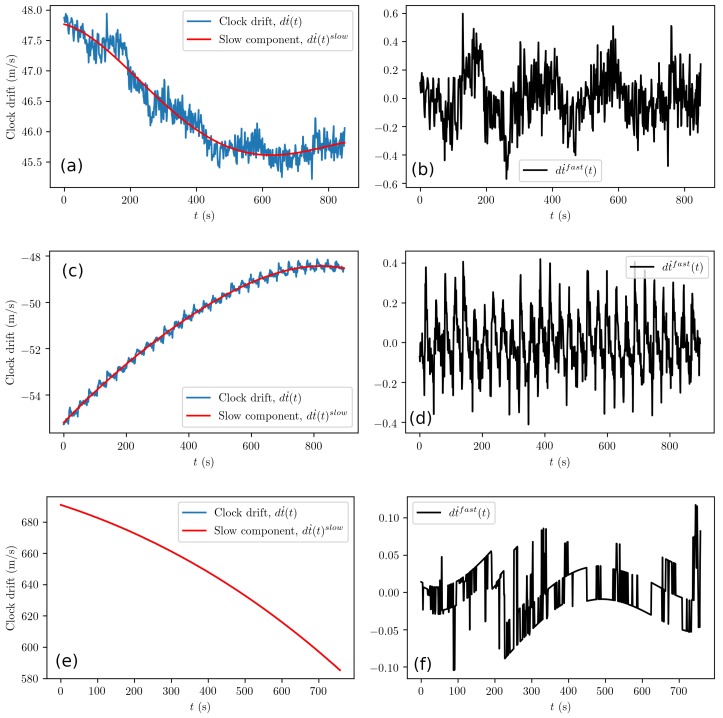
Open sky results for the (low-grade) Samsung S8 smartphone receiver (**a**,**b**), the (medium-grade) u-blox receiver connected to the patch antenna (**c**,**d**) and from the (high-grade) Septentrio receiver connected to the Leica antenna (**e**,**f**). In (**e**), clock drift $d\dot{t}$ and slow component lines overlap because the fast component $d{\dot{t}}^{fast}=d\dot{t}-d{\dot{t}}^{slow}$ shown in (**f**) is very small. Note that the saw-edge lines in (**f**) are due to the self-correcting behavior of the (high-grade) Septentrio clock.

The clock drift is shared with all NCOs and therefore also with all correlators that align to incoming signals. Remember that in the Methods section, we left as an open question whether the slow and fast component of the clock drift in Equation (5) can be separated. In Figure 3a,c,e, the clock drift is clearly separable, $d\dot{t}=d{\dot{t}}^{slow}+d{\dot{t}}^{fast}$, even for the smartphone receiver. This means that our assumptions related to the combination of measurements and the respective clock model hold. Here, the slow component is represented with a fourth order polynomial, which are commonly used in Commercial Off-The-Shelf (COTS) receivers for similar numerical purposes.

**Figure 4:**
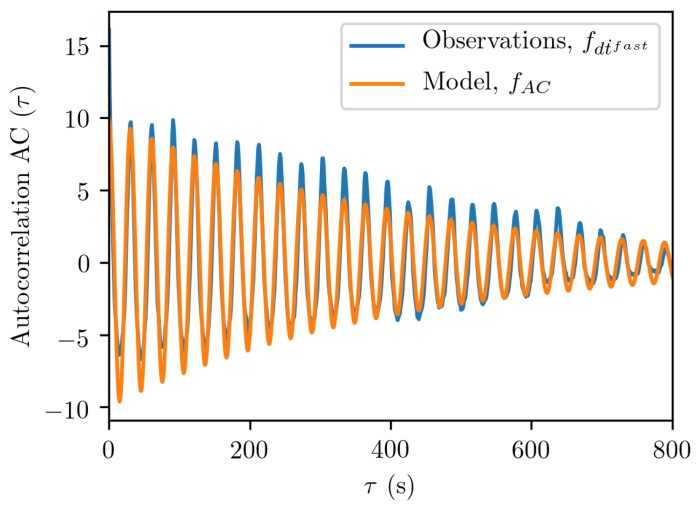
Autocorrelation function obtained from the clock drift $d{\dot{t}}^{fast}$ data and the related model function $f_{AC}$ of Equation (26), for the u-blox receiver connected to the patch antenna.

The fast component, which we were interested in, showed small 0.1–0.4-m/s noise in Figure 3b. These data, when without temporal correlations, can be modeled with a normal distribution, such as the rightmost term in Equation (7). The temporal oscillations of the fast component, however, required further analysis. Here, we performed an exemplary analysis of the u-blox data shown in Figure 3b, by calculating an autocorrelation function using Equation (24); see Figure 4. The autocorrelation function obtained from the fast clock drift followed the form of the following model function:

(26) $$f_{AC}\left(\tau \right)=ae^{-b\tau }cos\left(\omega \tau \right)$$

where the constants for the u-blox receiver with the patch antenna are a=10.0, b=0.0025, and ω=0.2070. In other words, the receiver clock exhibits a repetitive error that has a characteristic cycle of about 1.3 m/s, or dividing by the speed of light, a cycle of about 4.3 ns in the time domain. These oscillations that have a large magnitude were due to interference between the signal replica and the incoming signal, telling us about the free-running frequency of the crystal. Note that the autocorrelation function of Equation (26) for the clock drift is similar to, but simpler than the one predicted by Winkel for the DLL and PLL oscillations; see Equation (25). We shall see whether the DLL and PLL observations exhibit this same behavior. However, before we do this, we shall write out a clock offset model that encompasses both the random walk and the temporal correlations as follows:

(27) $$\begin{array}{c} \left\{\begin{array}{c} d{\dot{t}}_{k}=\frac{1}{a}\cos\left(\omega \;d{\dot{t}}_{k-1}\right)+\epsilon_{t_{k}}\\ dt_{k}=dt_{k-1}+\Delta t\;\;d{\dot{t}}_{k} \end{array}\right., \end{array}$$

where $\epsilon_{t_{k}}∼N\left(0,\sigma_{d\dot{t}}^{2}\right)$, *k* enumerates discrete time steps of length Δt, the constants *a* and ω that are GNSS receiver specific are obtained from Equation (26), and $\sigma_{d\dot{t}}^{2}$ that is also GNSS receiver specific is obtained from Equation (6). Note that $\sigma_{d\dot{t}}^{2}$ is equal to the variance of the fast component $d{\dot{t}}^{fast}$; see Figure 3b.

### 4.2. DLL and PLL Models

**Figure 5:**
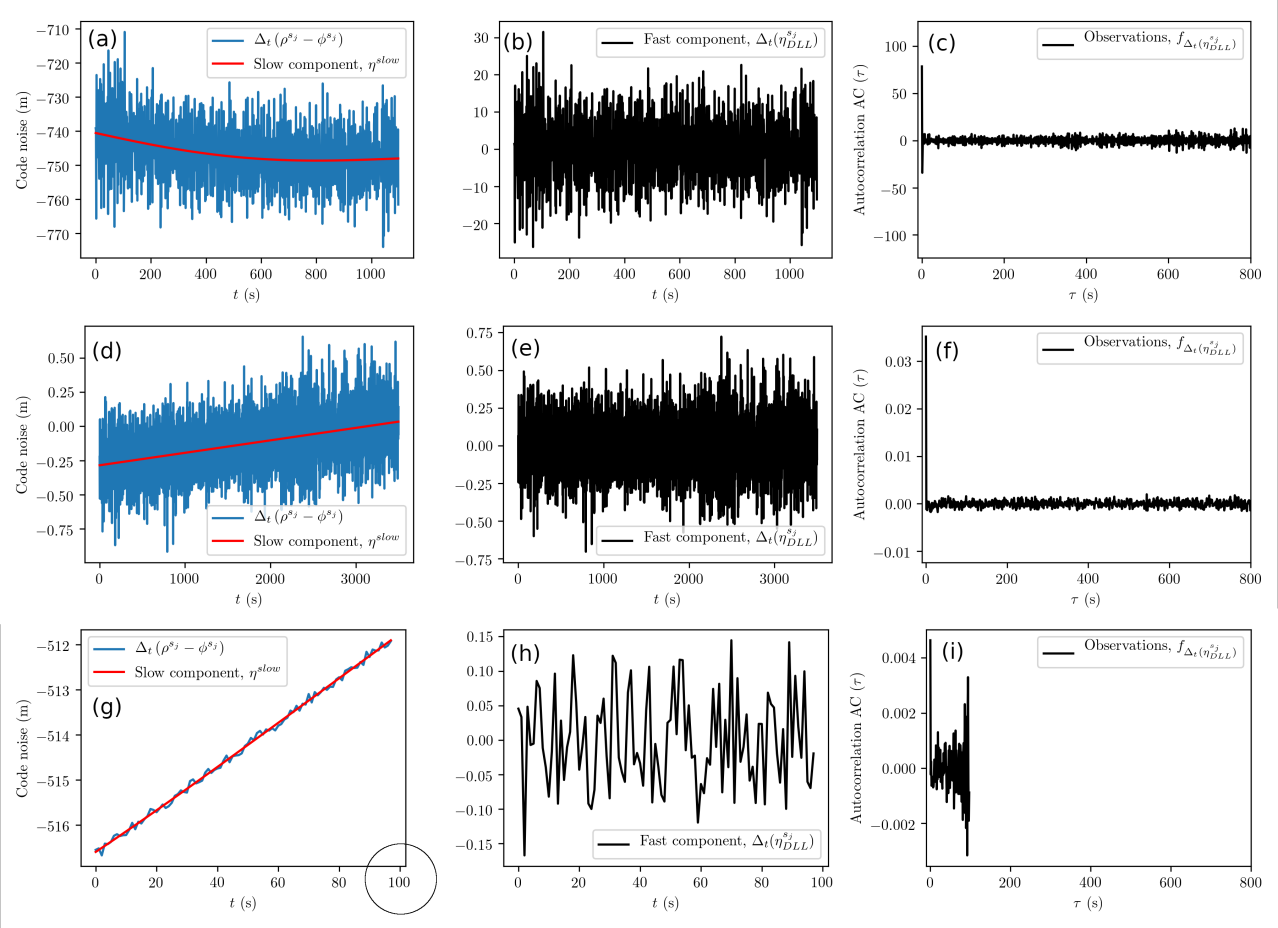
Code measurement noise analysis (Delay Lock Loop (DLL)) from GPS L1C pseudorange data for Samsung S8 (**a**–**c**), u-blox with the patch antenna (**d**–**f**), and Septentrio (**g**–**i**) receivers. (**a**,**d**,**g**) Raw data with a red line showing the slow component trend. (**b**,**e**,**h**) The fast component is within ±0.5 m. (**c**,**f**,**i**) Autocorrelation function calculated from the fast component. For visualization purposes, we have selected less data for (**g**); see the impact on (**h**) and (**i**).

In Figure 5, exemplary plots of the performed DLL error analysis are shown. The slow components, $\eta^{slow}$, appear linear, but are not; see Figure 5a,d,g. The subtraction of the slow component reveals the fast component shown in Figure 5b,e,h. The autocorrelation functions calculated from the fast component data, shown in Figure 5c,f,i, exhibit the temporal behavior of white noise. Therefore, we can conclude that the DLL error model of Equation (18) is adequate for simulation purposes. The variance of the normal distribution, $\sigma_{DLL}$, is obtained from the fast component, i.e., the time series shown in Figure 5b,e,h. In Figure 5b, the time series shows an up-down peak, i.e., an anomaly, that we conserved for visualization purposes. Otherwise, the times series used for the DLL and PLL analyses did not contain such anomalies and thus represented the lower bound for errors.

**Figure 6:**
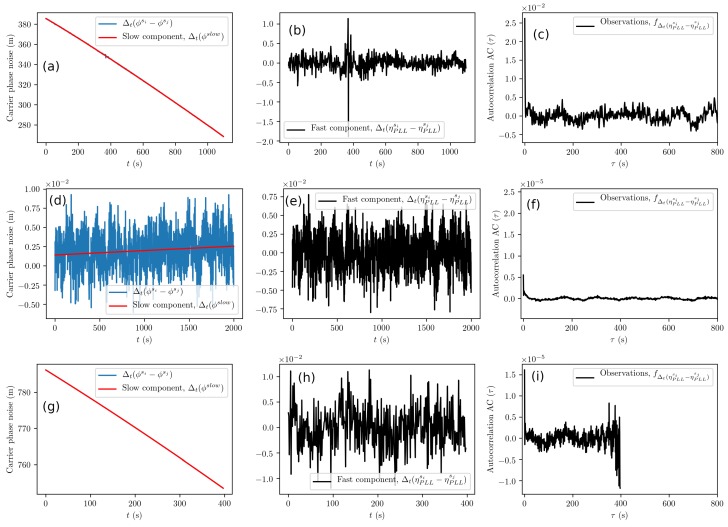
Carrier phase noise analysis (Phase Lock Loop (PLL)) from GPS L1C data for Samsung S8 (**a**–**c**), u-blox with the patch antenna (**d**–**f**), and Septentrio (**g**–**i**) receivers. (**a**,**d**,**g**) Raw data with a red line showing the slow component trend. (**b**,**e**,**h**) PLL fast component. In (**e**), variation is within ±7.5 mm. (**c**,**f**,**i**) Autocorrelation function calculated from the respective fast component. Note the anomaly in (**b**), most likely due to a loop slip. Furthermore, we selected a short times series in (**g**); see the impact on (**i**).

**Figure 7:**
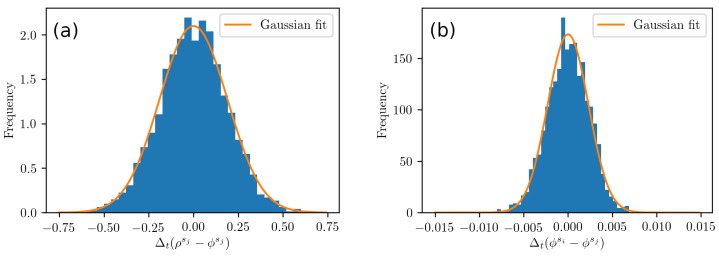
The distribution of the combination of measurements of Equations (17) and (21), in (**a**) and (**b**), testing whether modeling DLL and PLL errors, respectively, as Gaussian noise is appropriate. Data are from Figure 5e and Figure 6e.

The PLL noise in the carrier phase is analyzed using Equation (11), and exemplary results are shown in Figure 6a,d,g. For the u-blox receiver in (d), we can see that the phase noise is within ±1 cm. The fast component is shown in Figure 6e, and it is further analyzed by calculating the autocorrelation function; see Figure 6f. The autocorrelation function reveals no anomalous components. However, the validity of our assumptions requires further testing. The proposed PLL-related error model of Equation (23) employs a single normal distribution even though the combination of measurements of Equation (21) used to estimate its parameters uses data from two different satellites. Therefore, we examine the distribution of the measured observable (see Figure 7) and see that it is close to a Gaussian shape. Therefore, we conclude that using a normal distribution in Equation (23) is valid for simulating the phase noise caused by the receiver instrumentation.

### 4.3. Model Parameters for Different Receivers

Table 1 shows the results for the error parameters obtained from the experimental campaign. Huawei P10 did not track any Galileo satellites, and Samsung S8 tracked only one, so the appropriate columns are marked with Not Available (N/A). This happened despite the most suitable hour of the week being selected from the satellite almanac in terms of satellite visibility. A maximum of nine Galileo satellites were visible during the time of measurement, which is a large amount near Helsinki, Finland. From the results of Table 1, we can observe that the smartphones studied here, Huawei P10 and Samsung S8, have a clock of similar quality as the u-blox receiver. However, the smartphones suffer from DLL and PLL noise levels that are over one level of magnitude higher, and this is what makes them inferior in positioning compared to the u-blox receiver. One major cause for this is without a doubt the antenna, as the smartphones have only small integrated antennas.

The two u-blox receivers using separate antennas seem to output a similar level of noise; see Table 1. The difference in quality of the antennas should, however, be significant. The reason why the different quality of the antennas is not showing in the results is that the signal conditions were nominal. See the Discussion for future work.

The clock quality in the (professional) Septentrio GNSS-receiver is significantly higher than the clock quality in the other receivers due to the self-correction properties of the receiver time keeping; see the zigzag behavior or sudden “jumps” in Figure 3f. This same behavior makes the numerical estimation of the effective clock performance quite challenging, and therefore, this value in Table 1 is marked with (*), indicating only a very careful estimate and that it is highly likely that the clock performance was even better.

## 5. Discussion

Here, we studied smartphones with one of the most recent GNSS chipsets, i.e., multi-constellation single frequency receivers (GPS (L1), Galileo (E1), and GLONASS (L1)). However, our methodology that is based on the combination of measurements is straightforwardly applicable also to estimating the errors of multi-frequency smartphone receivers, e.g., Broadcom BCM47755 chip that has been on the market since June 2018 (Xiaomi Mi8 GPS (L1/L5), Galileo (E1/E5a), and GLONASS (L1/L2)).

The results of Table 1 offer the lower bound results for the clock, DLL, and PLL errors, meaning that if the GNSS signals become blocked or multipathed, the performances of the receivers obviously deteriorate from these. This lower bound can be seen to represent a feasibility threshold for crowdsourcing applications, i.e., if an application would require a higher performance than what is available in open sky conditions for certain equipment, that application is not feasible with that equipment. On the other hand, if the performance requirements for the feasibility are lower than what is the measured performance of certain equipment in open sky conditions, that equipment may contribute to the successful implementation of that application.

For DLL and PLL errors, we used the satellite signals with the highest signal to noise ratio, $C/N_{0}$. Even in environments where multipathing is present, such as in our semi-urban experiments, this signal is typically obtained from a straight line-of-sight observation. However, if the signal contains multipathing, the central assumptions for the models of Equations (18) and (23) are violated. This can be seen as a limitation of the proposed method. Furthermore, another limitation is that the quality of the time series with respect to this ratio can only be verified in the post-processing phase, so online measurements are not feasible. In the future, it would be interesting to establish the link between $C/N_{0}$ and the variance of the thermal DLL and PLL noise. The signal-to-noise ratio $C/N_{0}$ likely depends, e.g., on the satellite elevation angle and also on the antenna quality if signal receiving conditions are degraded.

In our study, the time series were at most one hour long. This enabled us to obtain coherent results from different measurement sites. However, if the statistical accuracy of these results would be increased by future work, the following should be taken into account. The slow and fast component separation done, e.g., in Equation (5) relies on the fitting of a polynomial function. Hence, if large time series are to be captured, the piece-wise behavior of these fitting functions should be guaranteed by using appropriate numerical tools, e.g., splines.

The receiver clock has an offset compared to the assumed nominal frequency of its crystal, which affects the measurements as formulated in Equation (1). Here, we studied this offset indirectly using Equations (6) and (7). This offset is not a constant, but an unknown function whose time development depends on the instrumentation properties and changes in the temperature. Temperature changes can occur in a receiver when the outside temperature changes, the receiver is heating up after start-up, and when the power consumption changes due to some part of the receiver becoming active and generating heat. A typical such part is the signal acquisition block that activates on a regular basis and usually consumes several mA of power.

If the receiver is prone to exhibit frequent cycle slips, the effect of these cycle slips are incorporated into the estimated (low-bound) error for the clock drift. If there are cycle slips, but these are rare, the clock data time series shows anomalous behavior, e.g., peaks. These “larger errors” come on top of the lower bound error we were attempting to estimate. In this case, as we analyzed the data semi-automatically, we saw and excluded this anomalous behavior. Automatic detection of anomalous behavior is a part of future work.

The behavior of the receiver clock offset is unique for each receiver type, as also noted in [21]. We modeled the clock offset with random walk. Temporal correlation analysis by using an autocorrelation function, as we did, see Equation (24), is also plausible. Another way to model the clock offset would have been, e.g., using the position LSE [21], instead of the velocity LSE, but this has several shortcomings for which we list the two major ones. First, atmospheric corrections would have had to be introduced, making the proposed modeling method less generic. Second, the residual error from these atmospheric corrections would have been impossible to separate from the instrumentation-based clock offset noise.

## 6. Conclusions

In order to assess the feasibility of a GNSS crowdsourcing application, the level of measurement errors that originate from GNSS receivers must be estimated. For consumer-grade receivers that have integrated antennas such as smartphones, this is a real challenge, as the exact location of the antenna is unknown. Furthermore, the separation of errors originating from the receiver and those originating from the atmosphere is crucial for this kind of error analysis. Here, we laid out the equations for combinations of measurements needed to assess the level of quality of smartphone GNSS receivers, developed models on how these (low bound) errors can be simulated, and then performed field experiments to verify that the assumptions made for the proposed methods do hold. Using GPS L1, Galileo E1, and GLONASS L1 signals, exemplary parameter values for the proposed error models were presented for low-, medium-, and high-grade GNSS receivers. Our methodology is straightforwardly applicable also to multi-frequency GNSS receivers, including smartphones.

Based on our results, we conclude that the noise that originated from instruments and showed in code and carrier phase measurements, i.e., DLL and PLL, had negligible time-dependent components and can be modeled as white noise. Instead, the (effective) clock offset may additionally exhibit a time-dependent behavior that can be modeled with terms familiar in the literature, namely, with an exponential decay and a periodic component.

## Figures and Tables

**Table 1:** Table for the measured error model parameters in open sky conditions, from Equations (6), (17), and (21). Parameters are for the models in Equations (7), (18), and (23). See Figure 1 about the test setup. (*) See the text for details.

		GPS L1 (m)		Galileo E1 (m)		GLONASS L1 (m)	
Receiver	Clock $\sigma_{d\dot{t}}$ (m/s)	$\sqrt{2}\sigma_{\mathrm{DLL}}$	$2\sigma_{\mathrm{PLL}}$	$\sqrt{2}\sigma_{\mathrm{DLL}}$	$2\sigma_{\mathrm{PLL}}$	$\sqrt{2}\sigma_{\mathrm{DLL}}$	$2\sigma_{\mathrm{PLL}}$
Huawei P10	0.38	8.32	0.31	N/A	N/A	15.51	0.16
Samsung S8	0.18	9.09	0.16	9.25	N/A	12.81	0.16
u-blox Patch	0.14	0.31	0.006	0.20	0.006	0.32	0.008
u-blox Leica	0.19	0.30	0.006	0.23	0.006	0.20	0.006
Septentrio	0.03 *	0.07	0.004	0.04	0.004	0.11	0.005
